# Spontaneous head movements support accurate horizontal auditory localization in a virtual visual environment

**DOI:** 10.1371/journal.pone.0278705

**Published:** 2022-12-06

**Authors:** Andrea Gulli, Federico Fontana, Eva Orzan, Alessandro Aruffo, Enrico Muzzi

**Affiliations:** 1 HCI Lab, Department of Mathematics, Computer Science and Physics, University of Udine, Udine, Italy; 2 Otorhinolaryngology and Audiology, Institute for Maternal and Child Health IRCCS “Burlo Garofolo”, Trieste, Italy; The University of Electro-Communications, JAPAN

## Abstract

This study investigates the relationship between auditory localization accuracy in the horizontal plane and the spontaneous translation and rotation of the head in response to an acoustic stimulus from an invisible sound source. Although a number of studies have suggested that localization ability improves with head movements, most of them measured the perceived source elevation and front-back disambiguation. We investigated the contribution of head movements to auditory localization in the anterior horizontal field in normal hearing subjects. A virtual reality scenario was used to conceal visual cues during the test through a head mounted display. In this condition, we found that an active search of the sound origin using head movements is not strictly necessary, yet sufficient for achieving greater sound source localization accuracy. This result may have important implications in the clinical assessment and training of adults and children affected by hearing and motor impairments.

## Introduction

Auditory localization is key for personal safety and effective everyday listening; a moving vehicle or an approaching person is often first identified by listening and then looking at the source. Knowing where to listen furthermore improves awareness of the surrounding environment, speech perception, and sound source identification in presence of multiple sources [1, 2]. Studies of human auditory localization made under various listening conditions have been ongoing for more than a century. Rayleigh [3] showed that the most important cues humans exploit to identify the position of an acoustic source in space are the interaural time difference (ITD) and interaural level difference (ILD), together combining in what is known as the Duplex theory. Despite their predominant role in horizontal localization, static binaural cues are not informative for vertical localization and front-back differentiation. Furthermore, they are also unable to resolve the spatial ambiguity caused by acoustic stimuli reaching the ears with the same ITD and ILD, commonly known as the cone of confusion phenomenon [4].

Wallach’s studies [5] suggested that dynamic ITD and ILD cues associated with small head movements play a central role in the resolution of front-back confusion of a sound source. The role of head movement in resolving this ambiguity has also been confirmed by other studies in which individuals were allowed, encouraged, or induced to move their heads during stimulus presentation [6, 7]. Moreover, in the case of a noise stimulus low-passed at 1 kHz, in which monaural spectral cues are lost, the front-back error rate was approximately 20% in restricted head movement condition, while decreasing to a significantly smaller percentage when head movements were prompted [8]. Dynamic cues elicited from head movements have been demonstrated to help localization in the vertical plane, too. Perrett and Noble [9] showed that, when high-frequency energy is absent from the acoustic stimulus or when normal external-ear function is distorted, elevation can still be assessed with horizontal head rotations (provided low-frequency energy is present in the signal). Sources in the upper-hemisphere can also be better distinguished from lower-hemisphere sources thanks to head rotation.

Head motion-induced dynamic cues are reduced to a secondary informative resource for horizontal localization if the front-back dimension is ignored. In experiments comparing stationary head listening with active listening in individuals who were tested either with ear open or occluded, head motion contributed to localization accuracy only when ear molds were worn [10]. In that study, a ceiling effect was hypothesized since accuracy in the open-ear condition was high even without head motion. To further stress the importance of head motion in spatial hearing, the reproduction of binaural stimuli based on head-related transfer functions (HRTFs) integrating dynamic information at different resolutions (5°, 2.5°, and 1°) resulted in greater localization accuracy than the reproduction of individually modeled HRTFs [11]. In spite of the large amount of research, a common principle underlying the different dynamic strategies of the head observed during sound localization has not been found. Experiments in which the head was left free to move showed that participants did not necessarily turn their heads in the direction of the sound, nor did they always move their heads to be able to localize the sound source with high angular resolution [12]. Head movement patterns differed among participants, suggesting that each individual acts according to a peculiar strategy [13]. These strategies include the selection and coordination of individual rotational and translational movements involving the position of the torso, head, and eyes [14].

The need for expressing the complexity and variability of such actions and postures using few motion parameters drove us to break down the spontaneous movement of the head into one measure of its dynamism during exposure to an acoustic stimulus, and another measure that considers its final position as a goal point. Such a point is often taken into consideration in experiments where the position of the head is constrained by a chin rest, allowing only rotational movement [15]. Our analysis therefore made use of two measures of head movement: *head distance* and *head divergence*. The former was used as a measure of head dynamism in studies examining the spontaneous action caused by acoustic perception [16], and corresponds to the total distance traveled by the head during exposure to the acoustic stimulus. The latter describes the final listening position as the difference between the horizontal angle of the target source and the angle of horizontal head orientation. Moreover, we also took the pointing method and the amount and type of visual information available during localization into account, as well as the combined effect of these two factors [17]. The proposed experiment was in fact conceptualized after preliminary testing of a few experimental setups at the Institute for Maternal and Child Health IRCCS “Burlo Garofolo” employing different pointing methods.

We decided to test the possible role of head movement during a simple and widely known task such as horizontal localization. If the active search of a sound source plays a significant role in terms of localization accuracy also in such a task, then head movement would be considered as an important strategy humans can put into action in general, and not only during specific tasks such as vertical localization and front-back resolution. At this point, it would make sense to test whether stimulating hearing-impaired listeners to put the same strategy into action has a chance to result in a comparable improvement.

Motivated by the aim of showing that motor activity can be beneficial to listening, we investigated the use and contribution of head movement in localizing sound sources in a visual virtual environment providing no visual localization cues. For both design and practical reasons, virtual reality was preferred rather than hiding a speaker behind a curtain or similar arrangement. A head-mounted display (HMD) has been used during the test for head tracking data collection and for providing a virtual visual scenario containing uninformative yet familiar elements, i.e., a simple natural landscape. In this way, children and adults can be more engaged in the task than they would be if they watched a blank screen or a conceptual visual scenario lacking any interest [18]. In this design choice, we were supported by research reporting that the error is not significantly different in horizontal localization tasks conducted in real environments or in their virtual replicas [19].

We let the participants move their heads freely during the task, as they would have done in an everyday activity when faced with the need to locate a hidden sound source, while keeping the task of hand-pointing. In this way, we decoupled the movement of the head from the pointing task, thus collecting the related data without the influence of hand action.

## Materials and methods

### Participants

37 volunteers (12 males and 25 females, mean age: 31.95 ± 8.07 years) with no history of neurological disease participated in the experiment. They reported normal or corrected-to-normal vision. When questioned about hand dominance, all verbally declared that they were right-handed. A standard audiometric evaluation demonstrated a bilateral normal pure-tone hearing threshold in all the participants. In particular, we considered as normal hearing sensitivity an average threshold at 500, 1000, 2000, and 4000 Hz equal or below 10 dB Hearing Level.

The study, involving healthy volunteers undergoing non-invasive diagnostic tests, was performed in accordance with the ethical standards as laid down in the 1964 Declaration of Helsinki and its later amendments, and it was approved by the Institute for Maternal and Child Health IRCCS “Burlo Garofolo” (Trieste, Italy) under the project “Ricerca Corrente 05/21”.

### Conditions

**Fig 1:**
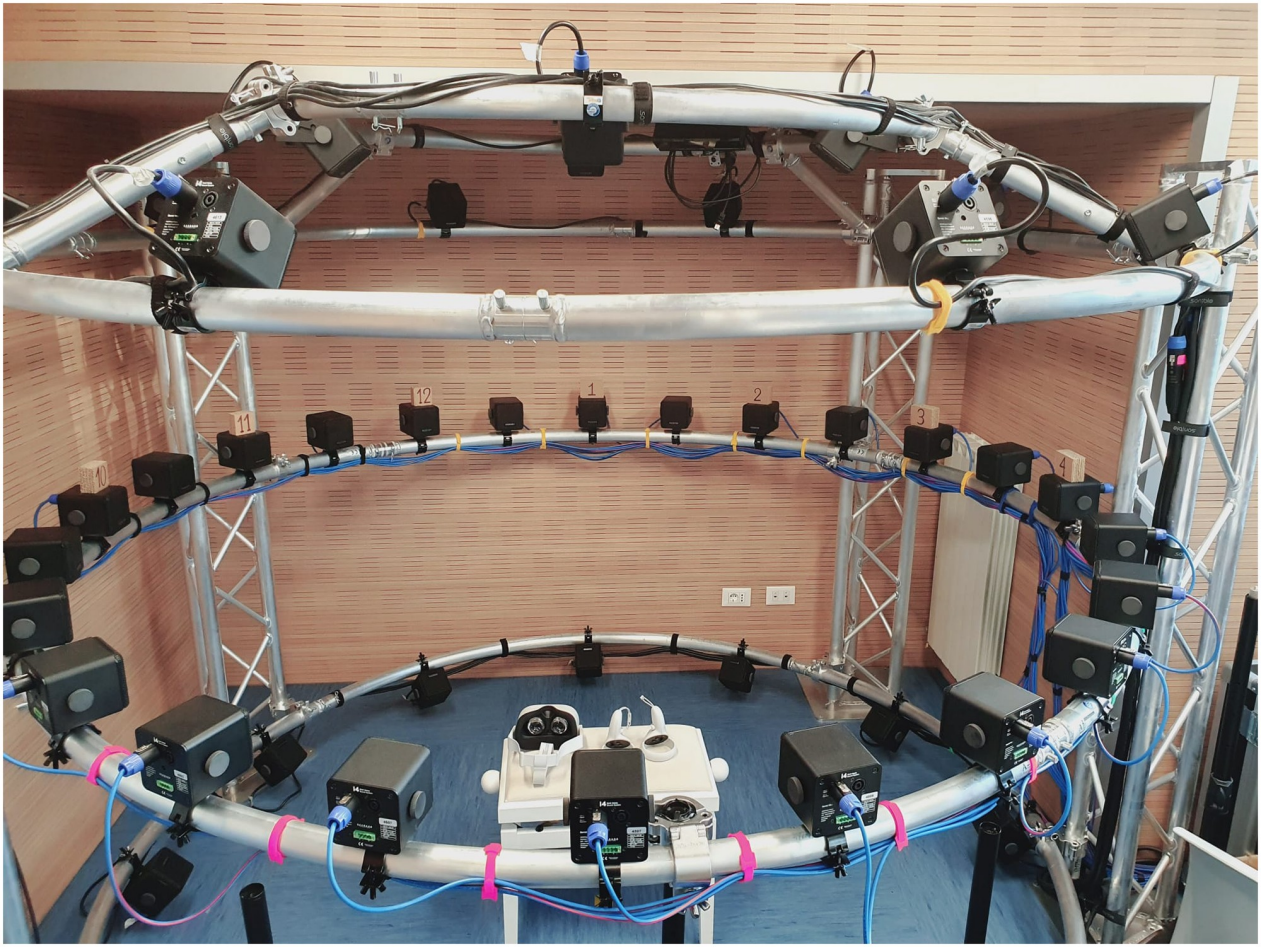
“La stanza di Matilde”: Institute for Maternal and Child Health IRCCS “Burlo Garofolo”s multifunctional audiological assessment space.

Two conditions, namely R (“real”) and V (“virtual”), were presented during a single session in randomly balanced order. In condition R, the control condition, participants could hear and see the array of loudspeakers in Fig 1 while wearing an HMD on the forehead in such a way that their vision was not occluded by the device. Participants pointed to the guessed sound source location with a laser pointer mounted on an Oculus Touch controller. In condition V, participants instead wore the HMD in an usual way, so as to be visually immersed in a virtual environment (VE), listening to the sounds coming from the loudspeaker array, and using the same controller to point towards the sound source. The VE provided participants with a homogeneous landscape, free of any absolute azimuth reference. In both conditions, participants were sitting on a height-adjustable seat, whose height could be adjusted by the experimenter.

### Setup

The acoustic reproduction system consisted of 13 Seeburg i4 loudspeakers (SEEBURG acoustic line GmbH) driven by a Sonible d:24 multi-channel amplifier (Sonible GmbH), part of the array visible in Fig 1 that is housed in a small enclosure sized 3 m x 2.6 m with a 60 dB-reverberation time RT60 = 200 ms. The loudspeakers were arranged in semi-circle, with a radius of 1.4 m. Using such an arrangement, 13 equally spaced horizontal directions of arrival were set up between −90° and + 90°, with an angular space equal to 15° between each pair of adjacent loudspeakers.

Navigation of the VE was enabled by an Oculus Quest 2 (Facebook Reality Labs, Meta Platforms), consisting of an HMD and two handheld controllers. The VE was developed in the Unity3D programming environment in collaboration with Isonlab s.r.l. The Oculus tracked the 3D position of the head with submillimeter precision [20] and the four-valued quaternion representing the orientation of the HMD with respect to the 3D position with a precision of ±1° at a 10 Hz sampling rate [21]. The data from the Oculus was received via the mqtt protocol [22] running on a 2.4 GHz Wi-Fi connection, by a mqtt broker as a Docker container (Docker, Inc.). From here, the data was sent to a custom client app, allowing the experimenter to monitor the head tracking and pointing systems as well as to check at runtime whether the connection between the Oculus and the computer was up and running, and whether the Max patch correctly synchronized the acoustic reproduction during the pointing task.

We aligned each virtual source with the corresponding loudspeaker by aiming at each speaker and then reading the angle displayed by the client app. When the app reported the correct target angle, we adjusted the physical laser to point to the center of the speaker. In this way, the participant was able to use the same controller and perform the same gesture to produce an identical response under both the R and V conditions. The resolution of the pointer angles was set to 1 degree, based on the Oculus controller accuracy rotation found in the literature [23].

### Stimuli

The acoustic stimulus consisted of a sequence of 200 ms-pink noise bursts, with onset and offset of 100 ms tapered linear ramps for each burst. The stimulus lasted until a response was produced. A new onset was separated by the previous offset by 200 ms of silence. A sequence of digital bursts at 16 bit with a 44.1 kHz sampling frequency was created using Max (Cycling ’74), and then sent to a MADIface USB 2.0 Audio Interface (RME GmbH). The stimulus was presented at a sound pressure level (SPL) equal to 65 dB ± 1 dB, measured with a calibrated sound level meter (XL2 Sound Level Meter, NTi Audio). This level was measured at the setup stage by placing the meter at the level of the experimenter’s external ear while he was seated in the test location. The measurement was repeated for each speaker and for both ears.

### Procedure

Before a participant started with a session, the seat was adjusted so as to place the loudspeakers at ear level. Then, the participants were instructed to use one controller with their dominant hand to point to where a sound source was located. At this point, they wore the HMD. The distance between the two HMD cameras was adjusted to each participant’s interpupillary distance, and the real and the virtual world were aligned through a spatial calibration procedure. Calibration was performed by asking each participant to point to specific visual markers occupying a play-area until an error of less than 1° was achieved for every loudspeaker. The resulting calibration was finally loaded through the Oculus Guardian system.

Before a test session started, each participant was asked to attend a brief training session including both the R and V conditions, during which they were acquainted with the task. In either condition, the training consisted of five hits. The test started with either the R or V condition, according to how the HMD was asked to be worn after the training. In both cases, the stimulus was presented by one loudspeaker over a randomly balanced sequence of 13×5 = 65 trials. Each trial ended when a point was hit by pulling the Oculus Touch trigger. At this very moment, acoustic stimulus stopped. Then, after pausing for one second in silence, a new trial began. We did not explicitly instruct the participants to respond as soon as possible.

Each participant was assigned to first test the R or V condition depending on his/her position in a randomly-balanced sequence of binary values. For the sake of compactness, we call *G*(*R*) the group that attended condition R first (17 participants, 46.06% of responses), and *G*(*V*) the other group.

### Data processing

From the recorded output of the system, we extracted the *signed error* as the difference between the target angle and the pointed angle [17]. From it, we computed the *unsigned error* as the absolute difference between the target angle and the pointed angle. This error was considered as a measure of overall accuracy. We also considered *latency* as the time taken by participants to localize one target [24]. Although the unsigned error is by its nature continuous, its measurement to the sexagesimal degree due to the precision of the Oculus controller has led us to analyze it with data bins 1 degree apart from each other.

From the 3D array of the positions and the 4D quaternion, we computed the difference between the target and head orientation angle in the moment when it was hit (*head divergence*), and the cumulative distance covered by the head during each trial (*head distance*).

Trials resulting in unsigned errors larger than 2 standard deviations above the mean deviation per target location were considered outliers and hence removed from our analysis [25]. Exclusion of the outliers is a common procedure in sound localization studies [17, 19]. The outliers finally were 123 out of 4810 trials, i.e., 2.55% of the total.

**Fig 2:**
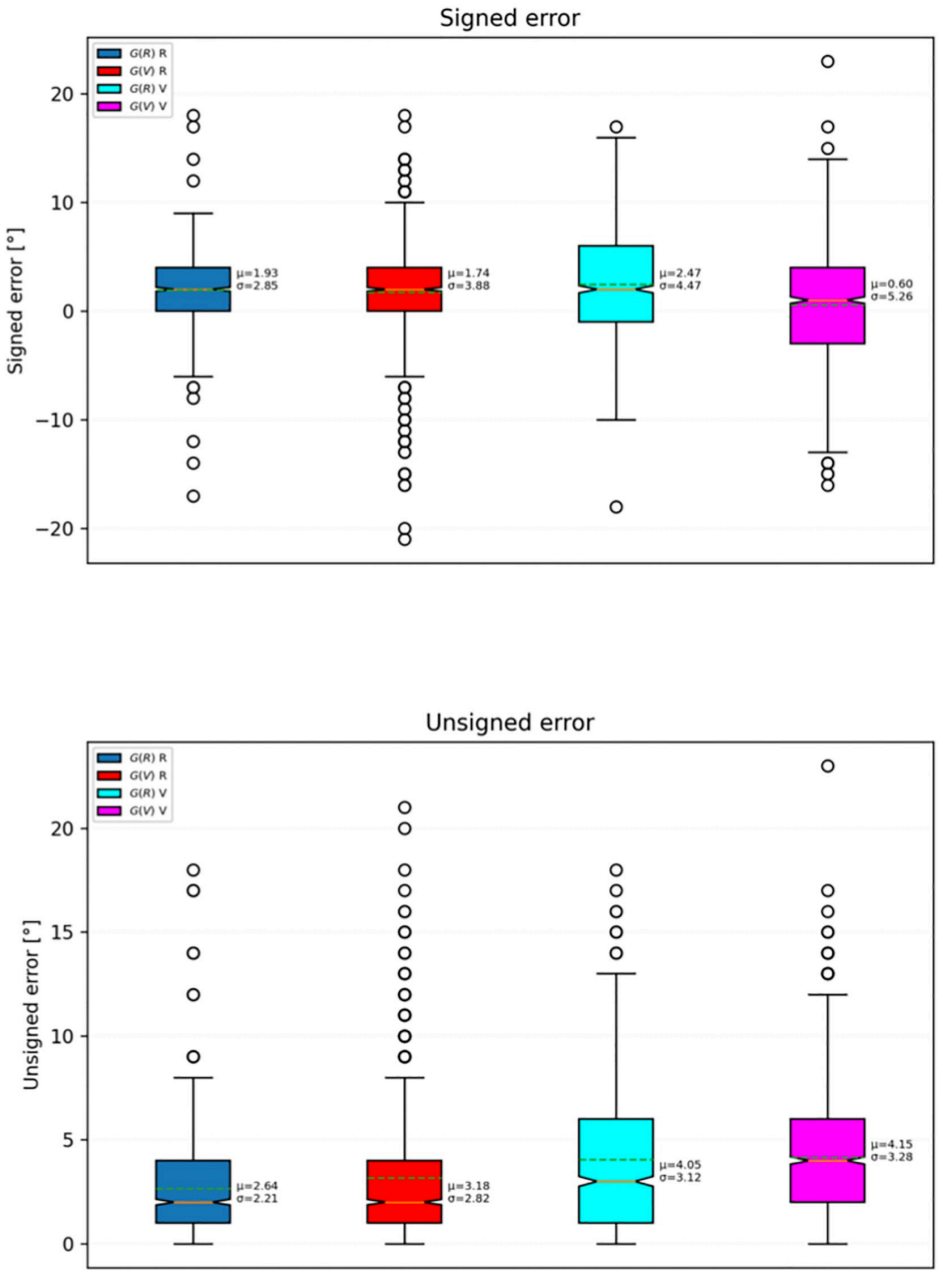
Errors by group and condition.

**Fig 3:**
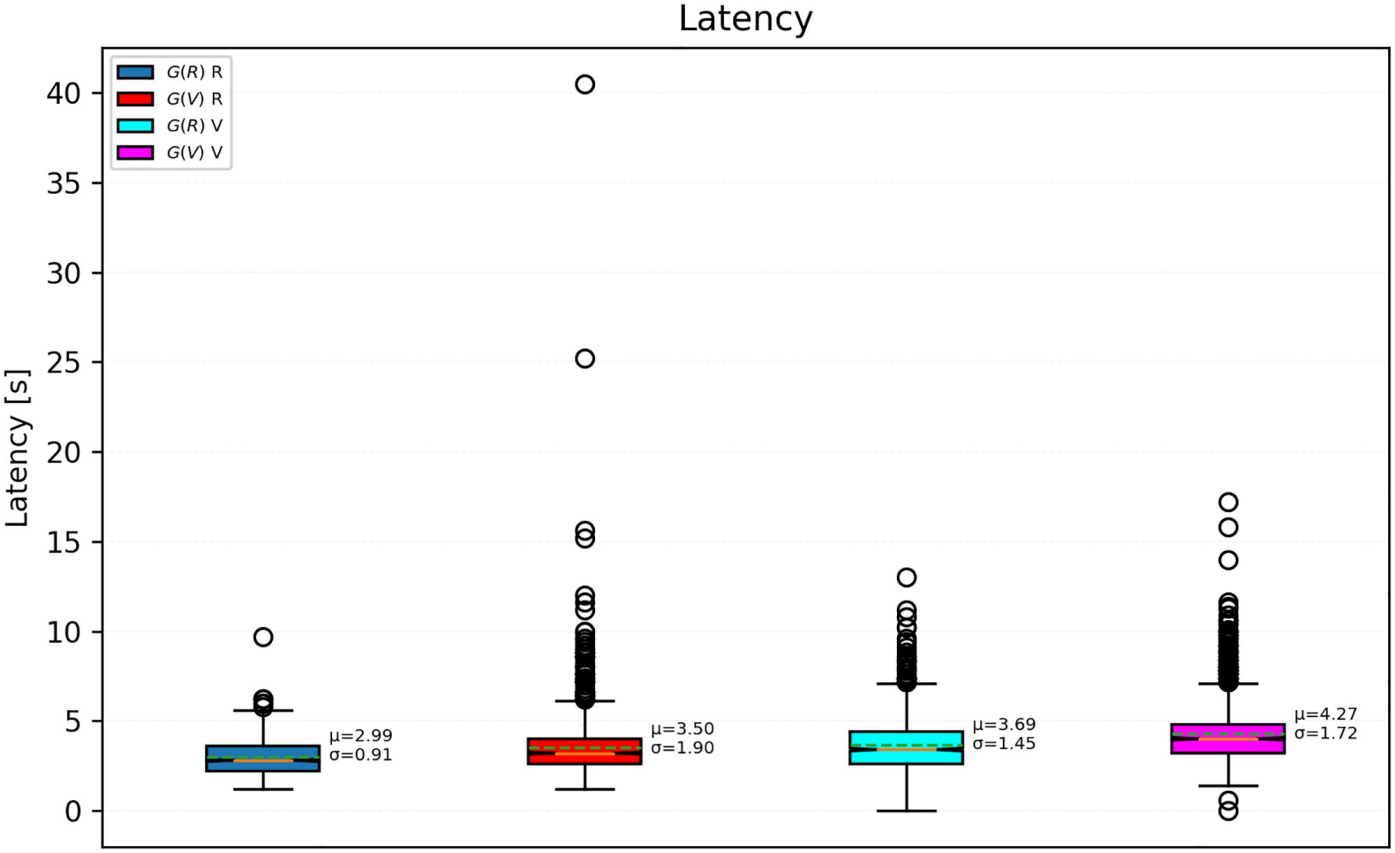
Response latencies by group and condition.

**Fig 4:**
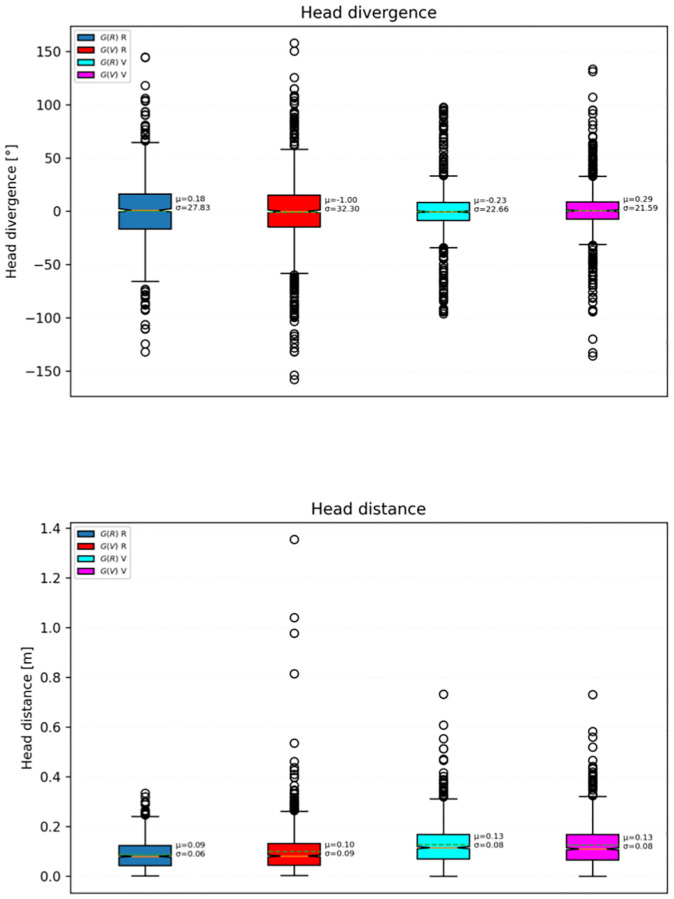
Head divergences and distances by group and condition.

**Fig 5:**
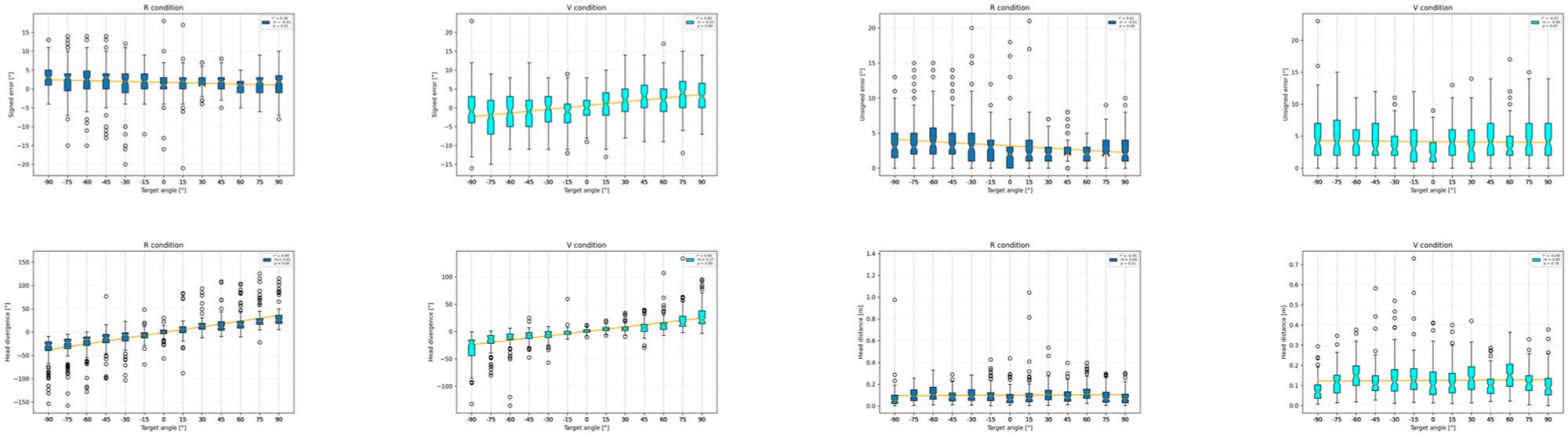
*G*(*V*) signed error, unsigned error, head divergence, and head distance as functions of the eccentricity of the target’s locations, with first-order regression curves fitted to the means per each target location.

Each dot in the plots presented in Figs 2–5, indicates a value in a single test. Except where otherwise noted, the data of all participants is pooled.

The statistical analysis started with Levene’s test to verify the homogeneity of variance among the analyzed groups. If the homoscedasticity hypothesis was met, we verified the equality of the means through the ANOVA F-value; otherwise, we used the one-way Welch ANOVA and Welch’s *t*-test. The statistical power was calculated for each test, and it was always found to be higher than 0.9. The correlation analysis was conducted with the computation of the Spearman correlation coefficient because we did not assume the normal distribution of the variables. All tests were two-tailed. In all multiple comparisons, Bonferroni correction was used. We considered the results statistically significant if the *p* value was less than 0.05. The statistical and correlational methods used in this paper were implemented by the Python package Pingouin [26]. The linear regression was done with Scikit-learn [27].

## Results

An analysis of both the variance (Levene’s test *W* = 16.24, *p* < 0.001) and mean (Welch’s *F*(1, 2346) = 87.13, *p* < 0.001) of the signed error in condition V showed a significant difference between the two groups (*σ*_*G*(*R*), *V*_ = 4.47°, *σ*_*G*(*V*), *V*_ = 5.26° and *μ*_*G*(*R*), *V*_ = 2.47°, *μ*_*G*(*V*), *V*_ = 0.60°). The smallest signed error mean was found in the V condition for group *G*(*V*) (*μ*_*G*(*V*), *V*_ = 0.60°), followed by the mean in the R condition of the same group (*μ*_*G*(*V*), *R*_ = 1.74°), the mean in the R condition of group *G*(*R*) (*μ*_*G*(*R*), *R*_ = 1.93), and the mean in the V condition of group *G*(*R*) (*μ*_*G*(*R*), *V*_ = 2.47°). All means are significantly different from one another (*p* < 0.001), except for the means in the R condition (Welch’s *F*(1, 2282) = 2.00, *p* = 0.16). The variances are all statistically different, too, (Levene’s test, *p* < 0.001) and the standard deviations are, in ascending order: *G*(*R*) in R condition (*σ*_*G*(*R*), *R*_ = 2.85°), *G*(*V*) in R condition (*σ*_*G*(*V*), *R*_ = 3.88°), *G*(*R*) in V condition (*σ*_*G*(*R*), *V*_ = 4.47°) and *G*(*V*) in V condition (*σ*_*G*(*V*), *V*_ = 5.26°). The unsigned error means (*μ*_*G*(*R*), *V*_ = 4.05°, *μ*_*G*(*V*), *V*_ = 4.15°) and standard deviations (*σ*_*G*(*R*), *V*_ = 3.12°, *σ*_*G*(*V*), *V*_ = 3.28°) in the V condition are both smaller for *G*(*R*). They are not statistically different from those of *G*(*V*) (Levene’s test *W* = 0.99, *p* = 0.32, ANOVA *F*(1, 2346) = 0.60, *p* = 0.44). The unsigned error means (*μ*_*G*(*R*), *R*_ = 2.64°, *μ*_*G*(*V*), *R*_ = 3.18°) and standard deviations (*σ*_*G*(*R*), *R*_ = 2.21°, *σ*_*G*(*V*), *R*_ = 2.82°) are significantly smaller for both groups in the R condition than their respective values in the V condition (Levene’s test, *p* < 0.001, and Welch’s *t*-test, *p* < 0.001). These results are graphically summarized in Fig 2.

Latency means are all significantly different from one another (Welch’s *t*-test, *p* < 0.001), although the difference is slightly less significant between the mean of *G*(*R*) in V condition and of *G*(*V*) in R condition (Welch’s *t* = 2.75, *p* = 0.006). They are, in increasing order: *G*(*R*) in R (*μ*_*G*(*R*), *R*_ = 2.99 s), *G*(*V*) in R, (*μ*_*G*(*V*), *R*_ = 3.50 s), *G*(*R*) in V (*μ*_*G*(*R*), *V*_ = 3.69 s), and *G*(*V*) in V (*μ*_*G*(*V*), *V*_ = 4.27 s). They are displayed in Fig 3.

There were no significant differences between the means of head divergence in conditions R and V, regardless of whether a participant began with condition R or V. Instead, for both groups, the mean distance of the head in condition V was significantly larger (Welch’s *t*-test, *p* < 0.001) than that found in condition R (*G*(*R*): + 44%, *G*(*V*): + 30%, see Fig 4).

Now, focusing on the group *G*(*V*) (20 participants, 1273 responses), the means of the signed errors grouped by their respective angles were fitted with a linear regression (*r*^2^ = 0.83, *m* = 0.03). Similarly, the head divergences across the same angles can be represented (see Fig 5) by linear regressions of their means across such angles respectively in the R (*r*^2^ = 0.99, *m* = 0.41) and V (*r*^2^ = 0.95, *m* = 0.27) conditions.

**Fig 6:**
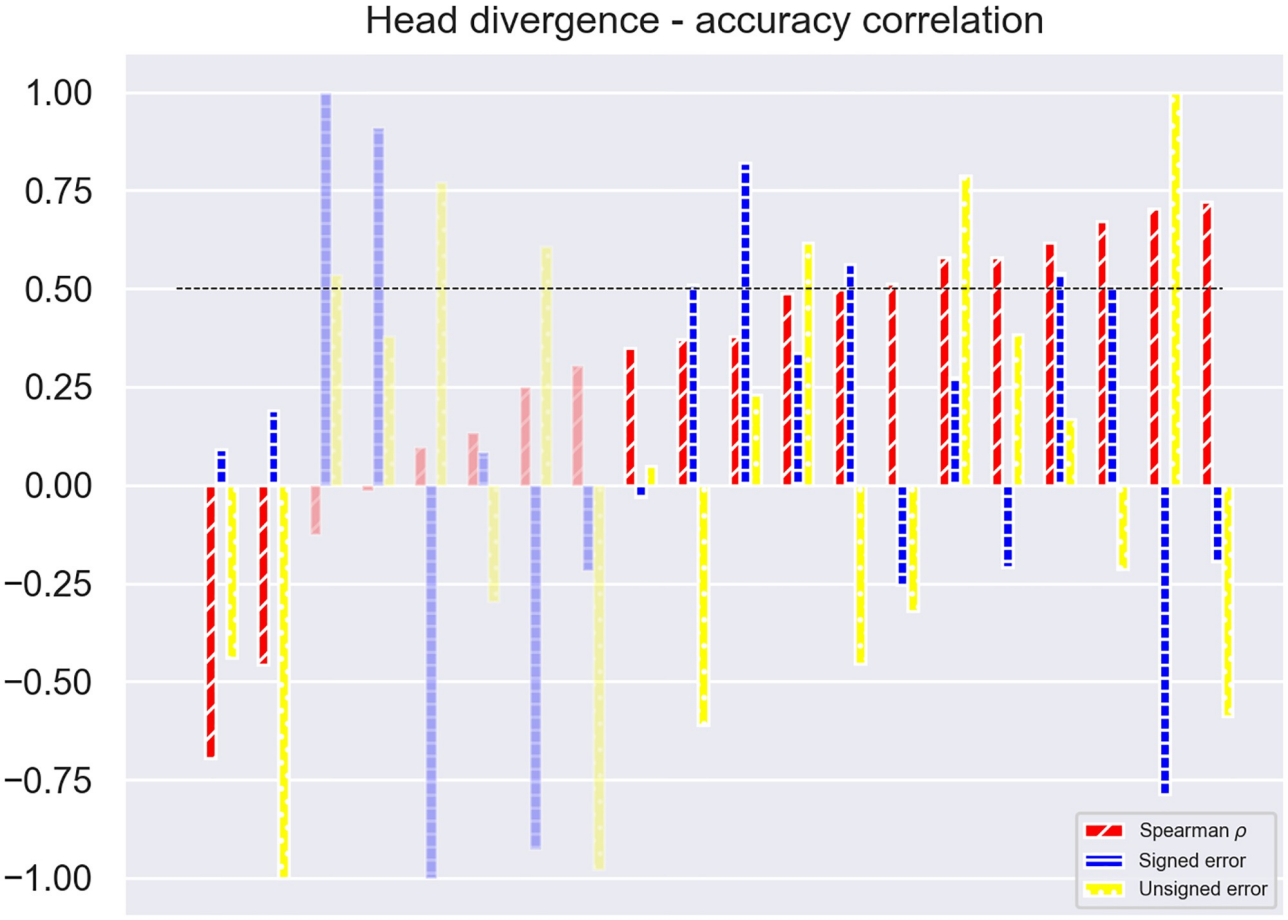
Spearman’s *ρ* correlation values (red) for each participant belonging to group *G*(*V*) in V condition, in ascending order, together with their respective means of the signed (green) and unsigned (blue) errors, normalized in the interval [-1, 1]. The opaque bars are the one with statistically significant correlations, while the transparent ones have *p*-values ≥0.05.

Partitioning the data across participants belonging to *G*(*V*) shows that a moderately positive correlation between the head divergence during the hit and the signed error exists for 7 individuals (Spearman’s *ρ* ≥ 0.5, *p* < 0.001). However, this sub-group is not homogeneous from a localization accuracy standpoint, as one can see in Fig 6. In the V condition, *G*(*V*)’s head divergence mean is 0.29°, and its standard deviation is 21.59°.

**Fig 7:**
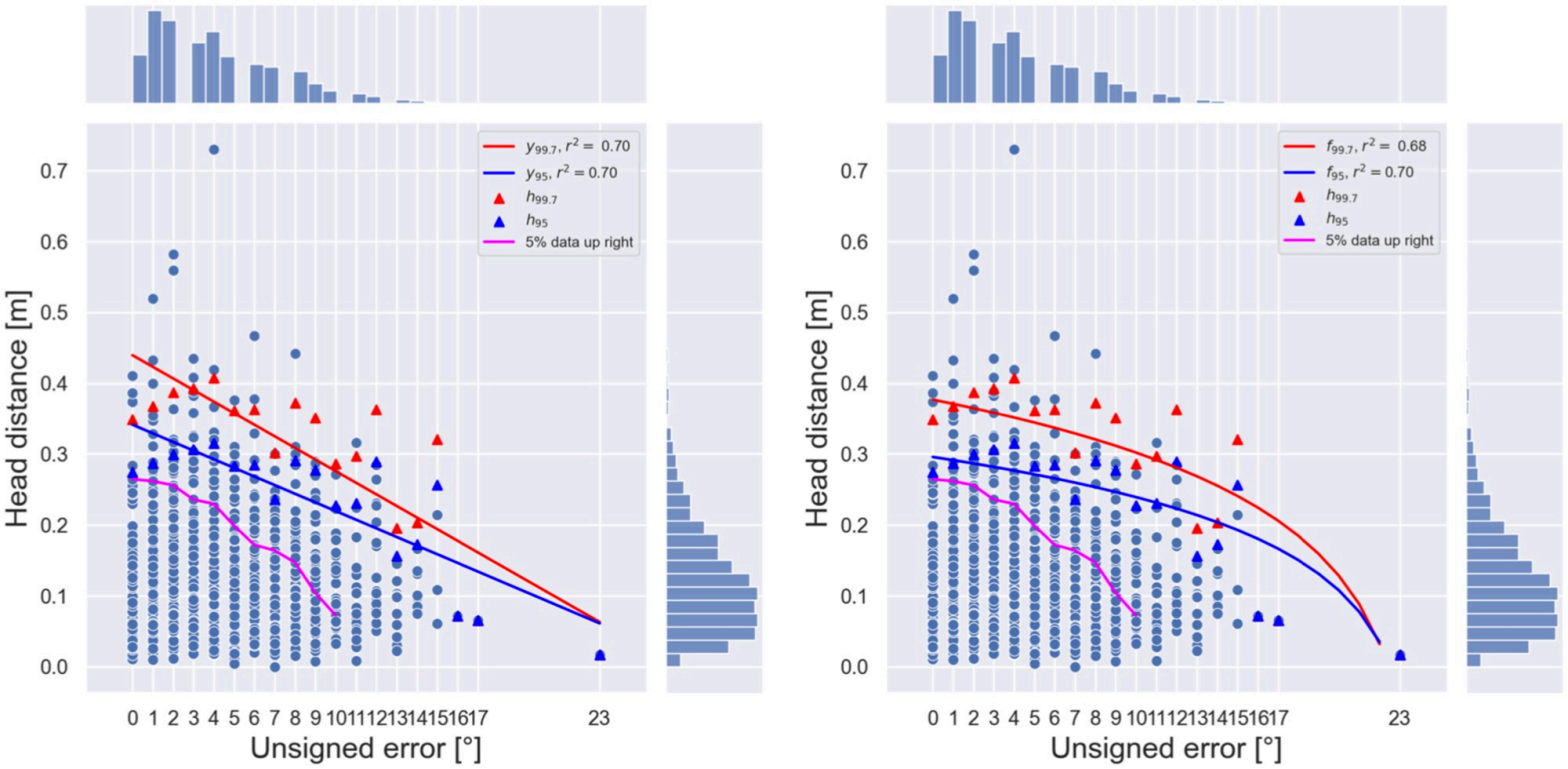
Head distance as a mapping of the aggregated unsigned error for group *G*(*V*) in condition V. Each dot indicates, respectively, the outcome of a single trial. Overlaid are the linear and logarithmic regression curves fitted to the 95% (blue) and 99.7% (red) confidence interval upper bounds, and the set of data points partitioning the plane in such a way that 5% of the entries are left in the upper-right quadrant cornered by the respective point (magenta). Each point on the “5% data up right” line is defined so that, taking that point as a lower left corner, the corresponding upper right quadrant contains 5% of the total number of data points.

For what concerns head distance instead, the aggregated data from group *G*(*V*) in condition V tends to fall into a *triangular* region if plotted as a function of the unsigned error (see Fig 7): the maximum values of such functions in fact decrease with the localization error. A linear regression of the 99.7% confidence interval upper bound (i.e., three times the standard deviation above the mean) across the head distance error, yields a coefficient of determination *r*^2^ = 0.705 and a slope *m* = −0.016. The computed linear regression is shown below:

$$h_{p,\epsilon }\approx m\epsilon +q,$$

where *h*_*p*, *ϵ*_ is the upper bound of the *p*%-confidence interval associated with the *ϵ* error level, *m* is the slope, and *q* is the intercept. Also, the upper bounds of the 95% confidence intervals of the head dynamic function decrease with increasing error levels.

A logarithmic regression gave similar results for both confidence intervals. In fact, we found a coefficient of determination *r*^2^ ≈ 0.7. Having then found two pairs of parameters *A*_*p*_ and *B*_*p*_ with the fitting of a logarithmically transformed linear regression such that

$$h_{p,\epsilon }\approx A_{p}\text{log}\left(\text{max}+1-\epsilon \right)+B_{p},$$

where *h*_*p*, *ϵ*_ is the upper-bound of the *p*%-confidence interval associated with the *ϵ* error level, and max is the maximum value of the unsigned error of group *G*(*V*) in condition V, we obtained two decreasing logarithmic maps, *f*_95_ and *f*_99.7_. The values of the parameters and the coefficients of determination of both the head distance confidence intervals regressions are described in Table 1.

**Table 1:** Linear and logarithmic regression descriptors for the 95% and 99.7% confidence intervals of the head distance.

	*p*%	*Slope*	*Intercept*	*r* ^2^
**Linear regression**	95	-0.012	0.342	0.697
	99.7	-0.016	0.440	0.705
	*p*%	*A*_*p*_	*B*_*p*_	*r* ^2^
**Logarithmic regression**	95	0.105	-0.038	0.698
	99.7	0.139	-0.064	0.682

The data points partitioning the plane such that 5% of the data remains in the upper right quadrant also define a decreasing function of the unsigned error. Each of these points is defined so that, taking that point as a lower left corner, the corresponding upper right quadrant contains 5% of the total number of data points.

## Discussion

The unsigned error distribution over the two groups and conditions confirms the existence of a learning effect in localizing the sound sources in the virtual environment on those participants who first attended condition R and then were tested when attending condition V [12, 28]. For all the participants, irrespective of belonging to *G*(*R*) or *G*(*V*), when speakers are visible in condition R then accuracy increases by approximately 1°.

The signed error is positive for both groups and both conditions, suggesting the existence of a localization bias that does not depend on vision. This finding corresponds to a rightward shift from the source position, a result which is in line with previous studies reporting that a rightward bias exists independent of which the dominant hand is and which hand is used during pointing [29, 30]. Moreover, there is an increase in the mean rightward shift occurring when participants attended the second condition, amounting to 1°. Hence, one may hypothesize this bias to be an effect of fatigue, too. This hypothesis is supported by the response latency if we interpret it as a measure of the effort required to perform the task. Although we did not explicitly instruct participants to respond as soon as possible, the task may have implicitly prompted participants to respond quickly as the acoustic stimulus was ongoing until a response had been received. However, participants were instructed to just hit the loudspeaker with the laser pointer when attending the R condition; this gave them freedom to miss the center of the speaker by some degrees while still completing the task successfully.

If the data from group *G*(*V*) is partitioned across angles, an undershoot effect is found confirming previous findings [17, 31] that signed errors tend to increase as a function of target eccentricity (see Fig 5).

The correlation between the head divergence and the signed error during the hit found in some of the participants’ data is probably connected to the pointing method and related gesture, which is reflected by the static function of the head rather than the dynamic one. Such a correlation may be explained as a substantial equivalence between the two acts of pointing with the controller and pointing with the head during a horizontal localization task, a result which has been repeatedly reported in the literature [17, 32, 33]. Although not asked by the experimenter, some participants in fact turned their heads towards the position they pointed with the controller.

The relationship between head distance and unsigned error suggests movement to be a sufficient condition for accurate localization. The absence of data points in the upper right quadrant in each plot of Fig 7 in fact tells that head movement is not necessary to ensure accurate horizontal localization; at the same time, it tells that head movement implies success in this task. The decreasing regression functions of the extension of the region where the unsigned error occurs with high probability suggest a proportional role of head motion in horizontal localization accuracy. It is tempting here to hypothesize the existence of a more general law behind this phenomenon, mapping head movement into localization accuracy similarly to what happens, e.g., in those hand-pointing tasks where the distance and area of a target correlate with the time to hit the target itself [34]. Such a law can only be speculated at this stage, as a substantially different experimental follow-up should be planned involving many more participants.

**Fig 8:**
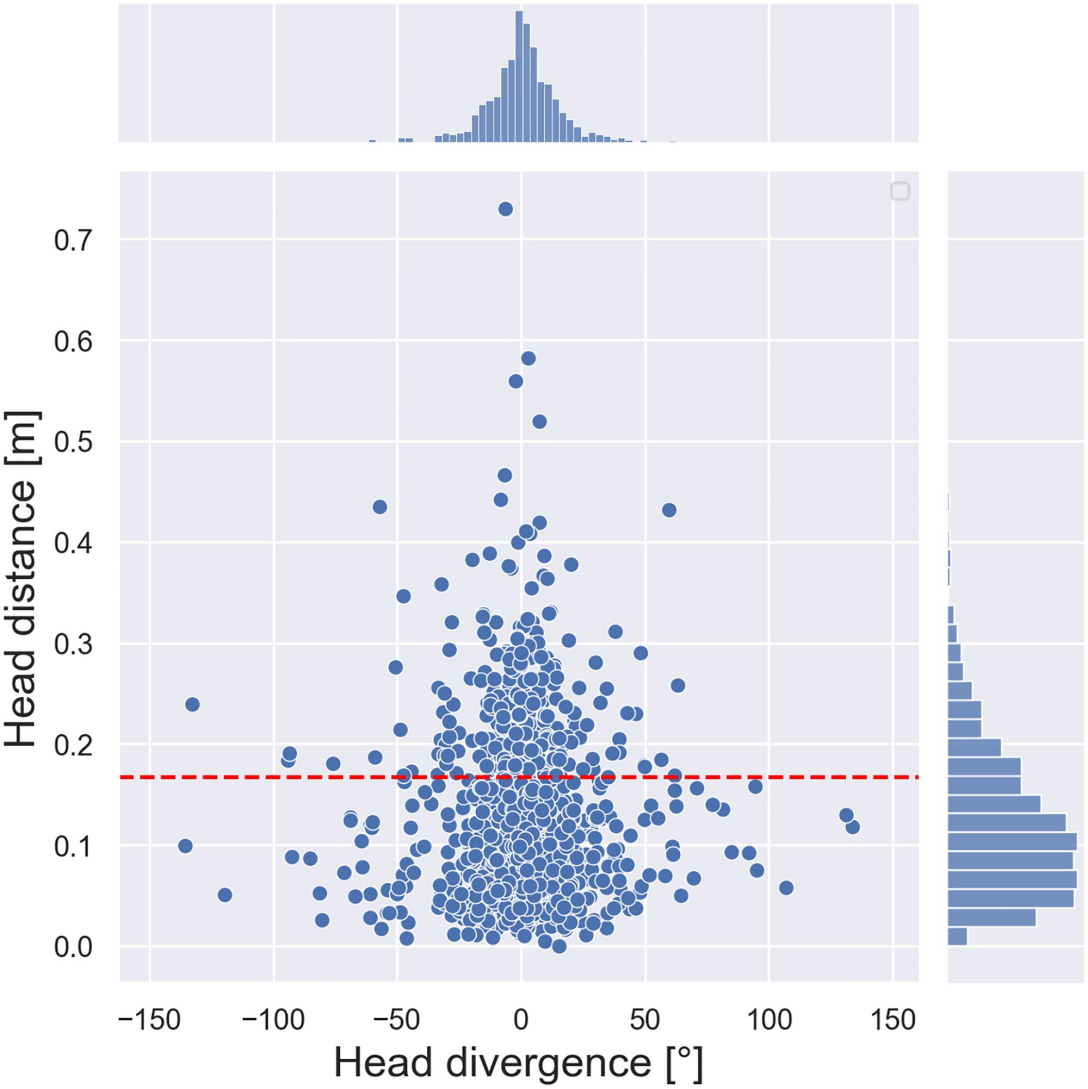
Head distance as a mapping of the difference between the target and head orientation angle during the hit, for group *G*(*V*) in condition V. The dashed red line represents the third quartile of the head distance, approximately 0.167 m. Each dot respectively indicates the outcome from a trial.

If we consider the relationship between head position during the hit (represented by head divergence) and head distance, we observe that the trials showing greater head motion lie in the neighborhood of the zero head divergence, i.e., they are such that the head is more precisely oriented toward the source, as it can be seen in Fig 8. This observation is supported by the reduction of both the mean and standard deviation of the head divergence of the points above the third quartile compared to the points below this quartile. The mean is closer to zero by ≈15.41% (ranging ≈0.55° to ≈−0.46°), and the standard deviation is reduced by ≈4.77% (ranging ≈21.84° to ≈20.80°). From this observation and from the fact that some participants’ responses showed a stronger correlation between the signed error and the head divergence, we speculate that, at least in some cases, an alternative strategy was used to localize the source, likely in connection with a higher confidence to hit the target when the head was oriented toward it. In general, individuals choose a strategy with the aim of optimizing their external behavior in front of internal limitations [35]. Instead of assessing ITD and ILD cues, hence performing a *differential* measurement, our participants who furthermore actively moved their head performed the assessment in terms of a differential threshold auditory task through head motion. In support of this observation, the literature claims that the detection of threshold signals is among the most elementary perceptual tasks [35] and furthermore [36] reports a specific attitude of listeners in discriminating interaural phase differences more acutely when the lateral auditory image shifts toward the midline than at either side. Based on this assumption, the more dynamic participants in our group felt confident in hitting the target only after a dynamic search for an angle in correspondence of which they judged two monaural signals to be identical [37]. Moreover, the fact that the distribution of head divergence is centered fairly close to 0° suggests that some participants switched from a more difficult differential assessment to a decision of interaural similarity based on their subjective threshold of interaural difference. Nevertheless, the aforementioned existence of a ceiling effect may explain why our participants hit targets with great precision even with low head motion.

Besides the ceiling effect, further limitations may have affected our study such as the use of wideband pulsed stimuli. They are ideal for sound localization, but they do not have direct ecological meaning in our virtual scene [38]. In general, our experiment had to compromise between the duration of a session, the number of source positions, and the number of repetitions for each sound source to avoid annoyance and disengagement from the task. Finally concerning the HMD, first wearing it differently in the R and V condition may have introduced a proportional bias between such two conditions in the participants’ HRTF; second, positional offsets can be difficult to be detected in proprietary VR systems even when real-world markers are used for calibration, and this may have introduced a pointing bias in our test [19].

Together, these considerations stimulate yet unexplored questions about the role of head motion in hearing-impaired individuals. It is well known that hearing loss disrupts binaural integration of auditory input, which is considered mandatory for accurate sound localization [39]. Hearing aids and cochlear implants can restore hearing threshold and speech perception in most cases, but outcomes in terms of sound localization accuracy vary widely from patient to patient [38, 39]. Previous studies have found that hearing impairment and directional microphones are associated with an increased complexity of orienting behavior towards the target [40, 41], and it has been shown that hearing-impaired listeners with asymmetric hearing loss successfully make use of head movements to increase their ability in a speech-in-noise task [42]. Nevertheless, another study [43] showed that young normal-hearing listeners had difficulty in finding a beneficial head orientation. Several patient-dependent factors have an impact on sound source localization skills, but studies on adults and children show large amounts of variability across listeners even with similar backgrounds. There can even be changes in everyday listening behavior associated with hearing loss and hearing aid use [44]. Further research is needed to clarify all these aspects.

## Conclusion

The purpose of the present study was to investigate the role of head movement in horizontal acoustic localization. While previous literature has clarified this role in vertical localization and front-back disambiguation, our study provides a ground-truth element about a localization task, the horizontal one, where head motion has traditionally been considered unnecessary. Our results do not contradict this assumption; instead, they contribute to completing the picture of the broader role of head motion in auditory localization.

If observed from the opposite perspective, the same results motivate an investigation into those situations in which limited movement is accompanied by limited localization. The low values shown by the correlation analysis are probably due to the secondary role of head movement in horizontal localization. Individuals with normal hearing in fact do not necessarily enable head motion unless the localization task becomes non-trivial. In this regard, horizontal localization of a spectrally rich sound source is fairly easy for this population group. However, the proposed methodology, along with the use of virtual reality, gains further interest when talking about hearing-impaired individuals. For this group even horizontal localization can be challenging, and checking low head motion in the presence of insufficient auditory localization may lead to motor therapy protocols with significant benefit for their listening abilities.

The use of virtual reality in the acoustic localization test paves the way for future developments incorporating some level of gamification in the virtual environment, which could be beneficial for assessing as well as training the localization ability of hearing-impaired adults and children.
